# Entecavir and Hepatitis B Immune Globulin in Patients Undergoing Liver Transplantation for Chronic Hepatitis B

**DOI:** 10.1002/lt.23690

**Published:** 2013-07-29

**Authors:** Robert Perrillo, Maria Buti, Francois Durand, Michael Charlton, Adrian Gadano, Guido Cantisani, Che-Chuan Loong, Kimberly Brown, Wenhua Hu, Juan Carlos Lopez-Talavera, Cyril Llamoso

**Affiliations:** 1 Hepatology Division, Baylor University Medical CenterDallas, TX; 2 Liver Unit, Vall d’Hebron University General Hospital/Network Center for Biomedical Research in Hepatic and Digestive Diseases, Carlos III InstituteBarcelona, Spain; 3 Hepatology Service, National Institute of Health and Medical Research CRB3, Beaujon Hospital, University of Paris VIIClichy, France; 4 Division of Gastroenterology and Hepatology, Mayo ClinicRochester, MN; 5 Hepatology Section, Italian Hospital of Buenos AiresBuenos Aires, Argentina; 6 Liver Transplantation Group, Santa Casa Hospital ComplexPorto Alegre, Brazil; 7 Department of Surgery, School of Medicine, Federal University of Rio Grande do SulPorto Alegre, Brazil; 8 Division of Transplantation Surgery, Department of Surgery, Taipei Veterans General Hospital, School of Medicine, National Yang-Ming UniversityTaipei, Taiwan; 9 Research and Development, Bristol-Myers Squibb CompanyWallingford, CT

## Abstract

For patients undergoing liver transplantation (LT) for hepatitis B virus (HBV)–related liver disease, the current standard of care for preventing reinfection of the allograft is nucleoside analogue therapy combined with hepatitis B immune globulin (HBIG). Entecavir has demonstrated high efficacy and a favorable safety profile for chronic hepatitis B (CHB) treatment, but data for patients undergoing HBV-related LT are limited. This study assessed the safety and efficacy of entecavir combined with various HBIG regimens after CHB-related LT. In this phase 3b, single-arm, open-label study, 65 patients undergoing LT for CHB-related liver disease with an HBV DNA load <172 IU/mL at LT received entecavir (1.0 mg daily) for 72 weeks after LT. The primary endpoint was the proportion of evaluable patients (treated for ≥4 weeks) with virological recurrence (HBV DNA level ≥50 IU/mL) through week 72. Concomitant HBIG therapy was received by 64 of the 65 enrolled patients, and 44% of these patients received high-dose HBIG (any HBIG dose in the specified interval ≥10,000 IU). Through week 72, all 61 patients evaluable for the efficacy analysis had undetectable HBV DNA. The Kaplan-Meier estimate of patients without hepatitis B surface antigen (HBsAg) recurrence at week 72 was 0.9655. Two patients experienced a reappearance of HBsAg, but both remained HBV DNA^−^ until the last follow-up. The frequency and nature of adverse events were consistent with those expected for this patient population. Serum creatinine increments ≥0.3 mg/dL and ≥0.5 mg/dL occurred in 62% and 39% of the patients, respectively, and all of these patients received calcineurin inhibitor therapy. In conclusion, in this population of patients treated with entecavir after CHB-related LT, entecavir was well tolerated and effective in maintaining viral suppression, even in individuals who experienced a reappearance of HBsAg. *Liver Transpl 19:887–895, 2013*. © 2013 AASLD.

Chronic infection with hepatitis B virus (HBV) can result in progressive liver disease, hepatocellular carcinoma (HCC), and death in as many as 25% of hepatitis B surface antigen (HBsAg) carriers.^1^ In patients with end-stage liver disease, liver transplantation (LT) is often the only treatment option. In Europe and the United States, 5% to 10% of all LT procedures are due to liver disease associated with HBV infection.^2^

The risk of recurrent HBV infection after LT for chronic hepatitis B (CHB) can be reduced by more than 90% with treatment regimens consisting of hepatitis B immune globulin (HBIG) combined with nucleos(t)ide analogue (NUC) therapy.^3^,^4^ Thus, combined therapy has become the standard of care at many centers worldwide. The choice of NUC is a critical factor, however, in preventing recurrent infections. The efficacy of lamivudine, currently the most commonly used NUC in this patient population, is limited by the frequent emergence of lamivudine resistance.^5^,^6^ In patients with lamivudine-resistant HBV, the addition of adefovir may be a possible treatment alternative; however, adefovir therapy may not always adequately control the replication of lamivudine-resistant HBV.^7^,^8^ In addition, adefovir has been associated with potential nephrotoxicity during prolonged use.^9^ This may be particularly problematic after LT because impaired renal function is common in these patients as a result of other comorbidities and immunosuppressive therapy with calcineurin inhibitors.^10^

Antiviral therapy using potent NUCs with a high genetic barrier to resistance and a favorable safety profile holds promise as a preferred regimen against HBV recurrence after CHB-related LT. Entecavir has demonstrated high efficacy and a favorable safety profile in the treatment of CHB,^11^–^17^ but currently, limited data exist for entecavir after HBV-related transplantation.^18^–^21^ Thus, the aim of the current study was to assess both the efficacy and safety of entecavir treatment combined with the discretional use of HBIG for preventing virological HBV recurrence after LT.

## PATIENTS AND METHODS

### Study Design, Patients, and Antiviral Treatment

This was a phase 3b, single-arm, open-label study of NUC-naive patients and NUC-experienced patients undergoing LT for CHB-related liver disease (NCT00395018). Patients were treated with entecavir (1.0 mg daily), which was started on the day of LT and continued for 72 weeks. The 1.0-mg dose was selected because some patients were expected to be lamivudine-experienced. Any previous anti-HBV therapy was at the discretion of the investigator and was continued during screening. Concomitant HBIG therapy was discretionary per the standard of care at each study site. At the end of the study treatment (week 72 or early discontinuation), further treatment with commercially available anti-HBV therapies was administered at the discretion of the investigator.

Patients eligible for inclusion were male or female adults (16 years old or older) who were eligible for LT because of end-stage liver disease associated with CHB (detectable HBsAg at screening and for 24 weeks or more before screening) and who had an HBV DNA load <172 IU/mL (approximately 1000 copies/mL) according to polymerase chain reaction at the time of LT. Patients were required to undergo LT within 180 days of screening. Patients with a pre-LT diagnosis of HCC were enrolled only if there was no evidence of extrahepatic spread, the tumor was solitary and ≤6.5 cm in diameter, or there were not more than 3 nodules with individual diameters ≤4.5 cm and with a total tumor diameter ≤8 cm. Patients were excluded if (1) they had a coinfection with human immunodeficiency virus, cytomegalovirus, Epstein-Barr virus, or hepatitis C virus; (2) they had HCC requiring systemic chemotherapy; (3) they were recipients of an ABO blood group–incompatible organ or multiple organs; (4) they had undergone retransplantation; (5) they had a recent history of pancreatitis; or (6) the donor had a cold ischemia time ≥20 hours.

The study was conducted in accordance with the ethical principles of the Declaration of Helsinki and the regulatory requirements of all participating countries. Institutional approval was obtained at all clinical sites, and written informed consent was provided by all study participants.

### Efficacy Analyses

Patients who received at least 4 weeks of entecavir treatment were considered evaluable for the efficacy analysis. The primary endpoint was the proportion of evaluable patients with virological recurrence through week 72 according to a last observation carried forward analysis, and this was defined as a serum HBV DNA level ≥50 IU/mL (approximately 300 copies/mL). Secondary endpoints included the proportion of hepatitis B e antigen–positive (HBeAg^+^) patients with HBeAg loss or HBeAg seroconversion at week 72 and the proportion of patients with HBsAg loss, hepatitis B surface antibody (HBsAb) positivity, or HBsAg recurrence (among those with on-treatment HBsAg loss) at week 72. A post hoc analysis evaluated HBsAg concentrations before transplantation to examine whether individuals with a reappearance of HBsAg had higher initial HBsAg concentrations in comparison with those who remained HBsAg^−^. HBsAg concentrations were also determined whenever possible on the day of transplantation and/or at weeks 12, 24, 48, and 72.

### Safety Analyses

Safety was assessed through week 72 and included the incidence of on-treatment adverse events, serious adverse events, deaths, and treatment discontinuations due to adverse events, as well as the incidence of liver disease progression (defined as new-onset complications such as variceal hemorrhage, jaundice, ascites, hyperbilirubinemia, and encephalopathy) as reported by the investigator. Episodes of liver rejection and retransplantation through week 72 were also recorded. Alanine aminotransferase (ALT) flares (ALT levels >2 times the baseline and >10 times the upper limit of normal), serum creatinine elevations (increases ≥0.3 mg/dL or ≥0.5 mg/dL versus the baseline), total bilirubin levels, and prothrombin times were recorded at week 72 and during off-treatment follow-up.

### Assay Methodologies

HBV DNA was assayed with the Roche COBAS TaqMan HPS assay (lower limit of detection = 10 IU/mL). An analysis of M204I/V and L108M mutations in screening samples was performed with the INNO-LiPA HBV DR assay. HBsAg levels were quantified with the Roche Elecsys HBsAg II assay (lower limit of detection <0.05 IU). All analyses were performed at a central laboratory.

### Statistical Methods

It was anticipated that approximately 70 enrolled and treated patients would be needed to provide 60 patients treated for at least 4 weeks. A sample size of 60 evaluable patients provided 95% confidence for stating that the virological recurrence rate with entecavir was <20% if there were ≤5 patients with virological recurrence 18 months after LT or that the virological recurrence rate was <7% if none of the 60 patients experienced virological recurrence 18 months after LT. The estimates of 20% and 7% for the virological recurrence rate at week 72 were based on a study of LT patients treated with lamivudine plus HBIG or with lamivudine alone.^22^

Categorical variables are summarized as counts and percentages. For the primary endpoint, the 95% confidence interval is presented. The distributions of continuous variables are summarized with univariate statistics (means, medians, and standard errors of the mean). A last observation carried forward analysis was used for the assessment of efficacy endpoints. The proportion of patients without HBsAg recurrence through week 72 was assessed with a Kaplan-Meier analysis; the time to recurrence was calculated from the date of the first on-treatment HBsAg loss to the date of the first on-treatment HBsAg recurrence or the last dose date.

## RESULTS

### Study Population

**Figure 1:**
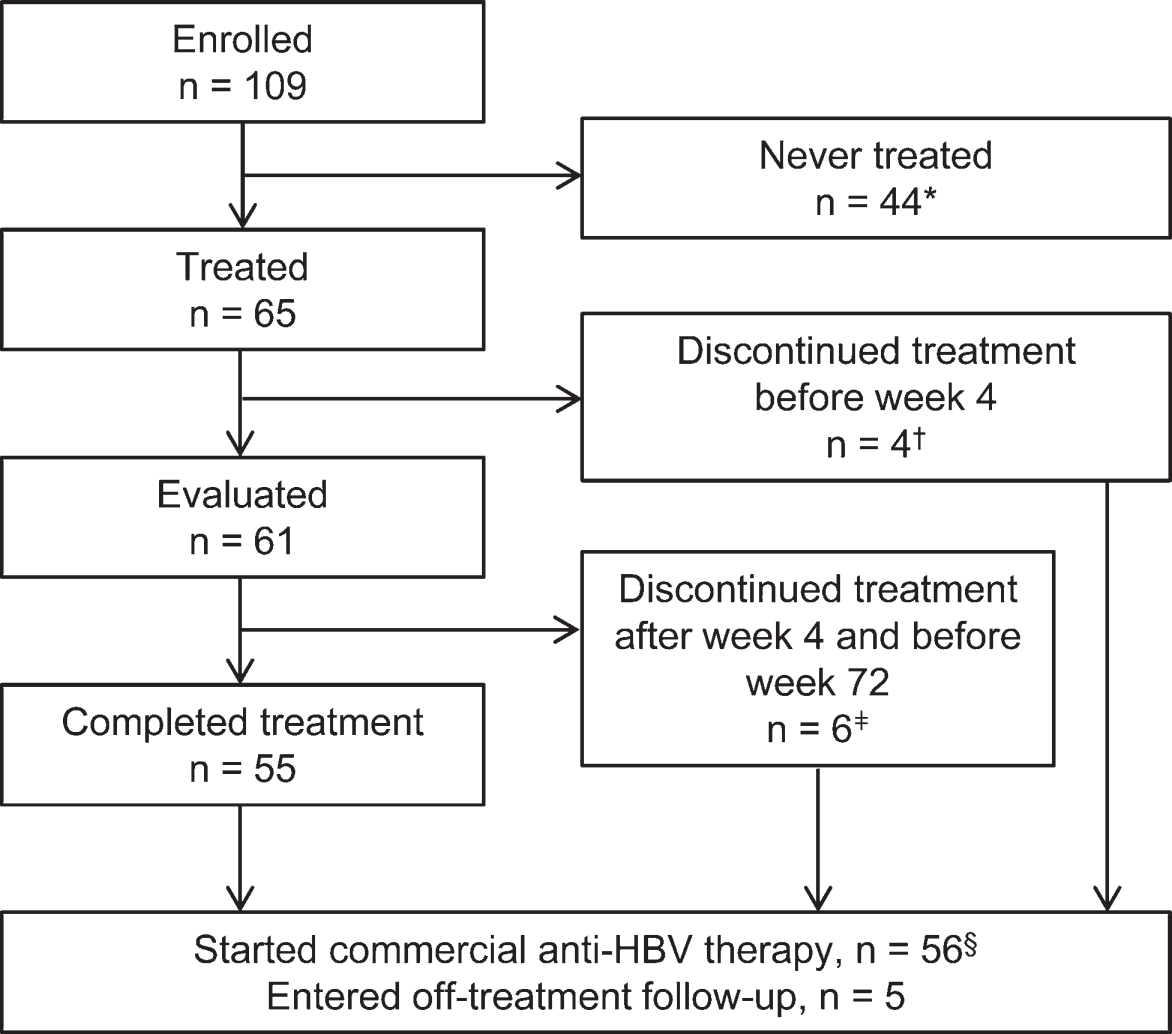
Screening, treatment, and follow-up of the study patients. *The reasons included the following: no longer meeting the study criteria because of changes in disease status and/or LT eligibility since the initial screening (n = 23), administrative reason by the sponsor (n = 9), withdrawal of consent (n = 6), death (n = 2), noncompliance (n = 1), and other (n = 3). †The reasons included the following: death (n = 2) and no longer meeting the study criteria (n = 2: one subject required retransplantation and was discontinued by the investigator, and the other subject had an HBV viral load > 172 IU/mL at the baseline). ‡The reasons included the following: death (n = 2), noncompliance (n = 2), and other (n = 2). §Entecavir (n = 53) and lamivudine (n = 3).

Twenty-seven study sites enrolled and treated patients: 8 in the United States; 3 each in Brazil, France, Italy, and Korea; 2 each in Australia, Spain, and Taiwan; and 1 in Argentina. One hundred nine patients were enrolled, 65 were treated with entecavir, and 61 received at least 4 weeks of entecavir treatment and were, therefore, considered evaluable (Fig. 1). Among the 44 patients who were never treated, the majority (23/44 or 52%) no longer met the study criteria because of changes in their disease status and/or LT eligibility since the initial screening (they did not undergo LT within 180 days of screening). Four patients stopped treatment before week 4, and another 6 patients discontinued the study before week 72. The reasons for discontinuation were death (n = 4), noncompliance (n = 2), no longer meeting the study criteria (n = 2), and other (n = 2). No patient discontinued the study treatment because of an adverse event. Fifty-five patients (90%) completed 72 weeks of entecavir therapy.

Demographic and pretransplant characteristics are depicted in Table 1. At the baseline, the mean age was 49 years, and the patients were mostly male (53/65 or 81.5%), HBeAg^−^ (58/65 or 89.2%), and HBsAg^+^ (61/65 or 93.8%). Prior antiviral therapy (with a single NUC or dual NUCs) was recorded for 81.5% of the patients (53/65) and included entecavir in 40.0% (26/65), lamivudine in 33.8% (22/65), adefovir in 18.5% (12/65), and tenofovir in 1 patient. The mean HBV DNA load at the baseline was 0.9 log_10_ IU/mL; all patients had an HBV DNA load <10 IU/mL at the time of transplantation. Mutant HBV DNA (M204I/V ± L180M) was detected at the baseline in 12.3% of the patients (8/65). The majority of the patients (38/65 or 59%) had normal or near normal ALT levels (≤1.25 times the upper limit of normal). Thirty-seven percent of the treated patients (24/65) had evidence of HCC in the explant. Among the 53 patients with pre-LT samples available for HBsAg testing, the mean HBsAg concentration was 1965 IU/L (range = 0.09-11,214 IU/mL).

**TABLE 1:** Baseline Demographics and Disease Characteristics of Subjects Treated With Entecavir (n = 65)

Age (years)*	49.3 (1.32)
Male [n (%)]	53 (81.5)
Race [n (%)]	
Asian	24 (36.9)
Black/African American	7 (10.8)
Native Hawaiian/other Pacific Islander	1 (1.5)
White	25 (38.5)
Other	8 (12.3)
Pre-LT anti-HBV therapy [n (%)]†	
Entecavir	26 (40.0)
Lamivudine	22 (33.8)
Adefovir	12 (18.5)
Tenofovir	1 (1.5)
Pegylated interferon	1 (1.5)
None	12 (18.5)
HBV DNA	
log_10_ (IU/mL)*	0.9 (0.06)
<10 IU/mL [n (%)]	65 (100)
M204I/V ± L180M mutations [n (%)]	
Present	8 (12.3)‡
Absent	22 (33.8)
Missing	35 (53.8)
HBeAg^+^ [n (%)]	7 (10.8)
HBeAb^+^ [n (%)]	48 (73.8)
HBsAg^+^ [n (%)]	61 (93.8)§
ALT (U/L)	
Mean (standard error)	160 (34.6)
Median	43
Albumin (g/dL)*	3.0 (0.08)
Total bilirubin (mg/dL)*	4.7 (0.78)
Tumors in removed liver [n (%)]	26 (40.0)
HCC∥	24 (36.9)
Type of transplant [n (%)]	
Cadaveric donor	54 (83.1)
Living donor	11 (16.9)
Donor age (years)¶	45.3 (5.0–85.0)
Cold ischemia time [n (%)]	
≥20 hours	4 (6.2)
<20 hours	50 (76.9)
Missing	11 (16.9)

NOTE: Four patients did not receive 4 weeks of entecavir treatment and were not included in the efficacy analysis. These patients were comparable to the overall patient group: there were 3 males and 1 female, the HBV DNA load was 0.78 IU/mL for 3 patients and 3.72 IU/mL for 1 patient, and no lamivudine resistance mutations were detected.

* The data are presented as the mean and the standard error.

† Monotherapy or combination therapy.

‡ Including 2 patients with M204I only, 2 patients with M204I and L180M, and 4 patients with M204V and L180M.

§ The 4 patients who were HBsAg^−^ on day 1 had been HBsAg^+^ on previous screening visits.

∥ Two tumors that were not diagnosed as HCC included a dysplastic nodule (1) and a benign focal lesion (1).

¶ The data are presented as the mean and the range.

### HBIG Therapy

HBIG regimens varied according to the standard practice at each site. All but 1 of the 65 treated patients (98%) received HBIG therapy at some point. Sixty of the 61 evaluable patients received HBIG. Data on the specific HBIG doses used each week and the means of delivery were not required for this study. HBIG dosing was arbitrarily classified as high if any HBIG dose in the specified interval was ≥10,000 IU and as low if the maximum individual HBIG dose during the interval was <10,000 IU. Changes in dosing occurred frequently. Table 2 shows the distribution of high and low dosing from day 0 to day 7, after day 7 up to 6 months, and more than 6 months after transplantation.

### Efficacy

**Figure 2:**
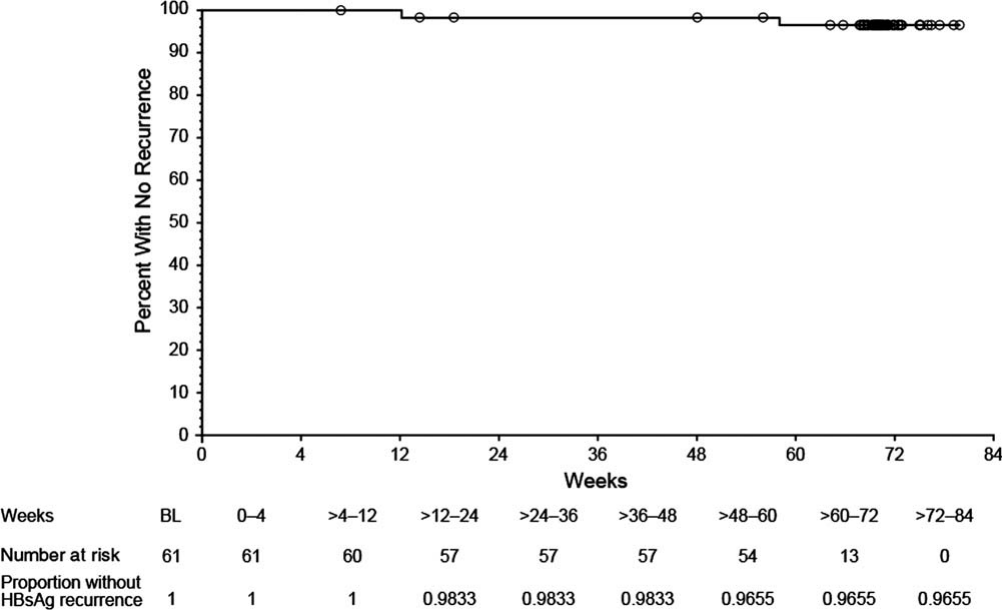
Kaplan-Meier plot of the proportion of evaluable patients without HBsAg recurrence. BL, baseline.

According to the last observation carried forward analysis, none of the 61 evaluable patients (0/61, 95% confidence interval = 0.0-5.9) had virological recurrence of HBV [HBV DNA level ≥ 50 IU/mL (equivalent to 300 copies/mL)] through week 72 (Table 3). Fifty-three of the 61 evaluable patients had HBV DNA measurements available for week 72, and no HBV DNA values were ≥50 IU/mL at the time of censoring for the 8 remaining evaluable patients. All 7 HBeAg^+^ patients (100%) lost HBeAg, and none had HBeAg seroconversion through week 72. HBsAg loss was sustained through week 72 in 59 of the 61 patients (96.7%), and 49 of the 61 patients (80.3%) remained HBsAb^+^. HBsAg reappeared in 2 of the 61 patients (3.3%; Table 4). The cumulative proportion of patients without HBsAg recurrence at week 72 was 0.9655 (Fig. 2). Neither of the 2 patients with HBsAg recurrence had detectable HBV DNA at the time of recurrence. Both patients were HBeAg^−^ and hepatitis B e antibody–positive (HBeAb^+^) throughout the study and HBsAg^+^ and HBsAb^−^ at the time of LT.

**TABLE 2:** Summary of HBIG Use in Patients Across the Study Time Period: The Evaluable Cohort (n = 61)

HBIG Dose [n (%)]	Days 0–7*	After Day 7 to 6 Months	After 6 Months
High dose†	28 (45.9)	24 (39.3)	21 (34.4)
Low dose‡	23 (37.7)	27 (44.3)	24 (39.3)
None	4 (6.6)	3 (4.9)	12 (19.7)
Dose not specified in IU	6 (9.8)	7 (11.5)	4 (6.6)
Patients Initially Using a High Dose [n (%)]	Days 0–7¢	After Day 7 to 6 Months	Patterns of Use After 6 Months
19 (31.1)	High dose	High dose	High dose
2 (3.3)	High dose	High dose	Low dose
2 (3.3)	High dose	High dose	None
1 (1.6)	High dose	Low dose	High dose
3 (4.9)	High dose	Low dose	Low dose
1 (1.6)	High dose	Low dose	None

* Day 0 was the day of LT.

† Any HBIG dose in the specified interval ≥ 10,000 IU.

‡ Maximum individual HBIG dose during the interval < 10,000 IU.

**TABLE 3:** Efficacy of Entecavir Through Week 72

Virological and Serological Efficacy Through Week 72	n/N (%)	95% Confidence Interval
Virological recurrence (HBV DNA ≥ 50 IU/mL)	0/61 (0)	0.0–5.9
HBeAg serology (among those HBeAg^+^ at the baseline)		
HBeAg loss	7/7 (100)	59.0–100.0
HBeAg seroconversion	0/7 (0)	0.0–41.0
HBsAg serology		
HBsAg loss	59/61 (96.7)	88.7–99.6
HBsAb^+^	49/61 (80.3)	68.2–89.4
HBsAg recurrence*	2/61 (3.3)	0.4–11.3

* Defined as HBsAg positivity after on-treatment HBsAg loss.

One of the patients, a 51-year-old Asian male, received concomitant HBIG therapy (1000 IU) for 5 days only. He became HBsAg^−^ and antibody to hepatitis B surface antigen–positive on day 28, and he subsequently became HBsAg^+^ again on day 433. The patient had no history of HCC. Before LT, he had received lamivudine and adefovir therapy for 20 months, and mutant HBV DNA (M204V and L180M) was detected at study entry. HBV DNA levels were below the limit of detection (<10 IU/mL) throughout the study. The HBsAg concentration before transplantation was 167.5 IU/mL.

The other patient, a 65-year-old white male who underwent LT for HCC, received 10,000 IU of HBIG therapy on day 1, and this was followed by 3 monthly doses of 4000 IU and 2000 IU monthly for less than 6 months. He became HBsAb^+^ on day 1 and HBsAg^−^ on day 27, and he became HBsAg^+^ again on day 111 and HBsAb^−^ again on day 167. HCC reappeared 6 months after LT and resulted in his death on day 252. The patient did not have any prior anti-HBV treatment. At the time of transplantation (day 1), he had an HBV DNA level <10 IU/mL with no evidence of lamivudine resistance mutations. After LT, his HBV DNA level was below the limit of detection (<10 IU/mL) through the time of HCC recurrence and death. The HBsAg concentration before transplantation was 2485 IU/mL.

### Safety

The frequency and nature of adverse events were consistent with those expected for this population. On-treatment adverse events occurred for 95.4% of the patients and were deemed to be serious in nature for 55.4%. The most common serious adverse events (>10%) were infections (16.9%), hepatobiliary disorders (15.4%), and gastrointestinal disorders (12.3%). The most frequent individual serious adverse events were acute renal failure and hepatic artery thrombosis (n = 3; all within 2 weeks after LT). All serious adverse events were of the expected nature and frequency for LT patients, and all were considered unrelated to the entecavir therapy by the study investigator. No patient discontinued treatment because of adverse events. ALT flares occurred in 9.2% of the patients (6/65), and all occurred within 2 weeks of LT. No ALT flare was associated with an increase in HBV DNA or acute cellular rejection, and all resolved within 1 month without complications.

Creatinine elevations ≥0.3 mg/dL and ≥ 0.5 mg/dL from the baseline occurred in 61.5% and 38.5% of the 65, respectively. All patients with increased levels of serum creatinine received calcineurin inhibitors as part of their immunosuppression regimen. Five of these patients had a history of diabetes. Events of liver disease progression were recorded in 15 patients (23.1%). Among these patients, 14 (21.5%) had ascites, 1 (1.5%) had spontaneous bacterial peritonitis, 1 had hepatic encephalopathy, and 1 developed HCC. All except 1 event (grade 1 ascites on day 240) occurred within 30 days of transplantation. Among all the treated patients, 5 deaths occurred. Four of these deaths occurred during treatment (at days 8, 12, 72, and 252). Causes of death included acute liver rejection (n = 1), septic shock/sepsis (n = 2), multiorgan failure (n = 1), and HCC recurrence with metastasis (n = 1). No death was considered related to the study medication by the study investigator.

**TABLE 4:** Clinical and Virological Features for 2 Patients With the Reappearance of HBsAg

	Patient 1	Patient 2
Baseline features		
Pre-LT NUC therapy	Adefovir + lamivudine, 20 months	None
Pre-LT HCC	No	Yes*
Mutant HBV (M204I/V ± L180M)	M204V + L180M	No
HBIG regimen	1000 IU for 5 days	10,000 IU on day 1, 4000 IU monthly (3 months), 2000 IU monthly
Time to HBsAg^+^ (days after transplantation)	433	111
HBV DNA (IU/mL)		
At first HBsAg^+^ reading	<10†	<10†
At the end of follow-up	<10†	<10†
HBsAg concentration (IU/mL)		
Before transplantation	167.5	2485
Day 1 after transplantation	131.3	312
Week 12	<0.05	<0.05
Week 24	Not available	27.92
Week 72	0.48	Not available‡

* Recurrence 6 months after LT.

† Lower limit of detection.

‡ The patient died 9 months after transplantation because of HCC recurrence.

## DISCUSSION

In the current study, 61 patients who underwent transplantation for CHB received entecavir in combination with an investigator-selected HBIG regimen. No patient experienced recurrence of HBV DNA positivity through week 72 of therapy, which was the primary endpoint of the study. The study was designed to assess the safety and efficacy of entecavir in the setting of the current standard-of-care management after LT (HBIG plus NUC). Although recently published studies have reported that HBIG can be safely withdrawn from patients maintained on NUC therapy with a high genetic barrier to resistance, the optimal timing of HBIG withdrawal and the predictors of a successful response with this approach have not been well studied.^23^,^24^ Moreover, most studies have involved relatively small numbers of patients or have not provided long enough follow-up to exclude late recurrence of infection. Thus, we believe that our study employing a continuation of HBIG with entecavir remains clinically relevant. Although our study involved a variety of HBIG regimens, this has been shown to reflect real-world practices.^25^

In the present study, 97% of the patients sustained HBsAg loss after LT. HBsAg reappearance was observed in only 2 patients and occurred 433 and 111 days after LT. One of these patients had lamivudine-resistant HBV detected before transplantation. Importantly, both patients remained HBV DNA^−^ at the last follow-up many months after the antigen had reappeared. Neither patient had unusually high HBsAg concentrations at the time of transplantation, and both initially became HBsAg^−^ a few months after transplantation.

It should be noted that the absence of detectable HBV DNA in patients with recurrent HBsAg positivity after transplantation has been described before in patients maintained on a combination of lamivudine and adefovir after HBIG withdrawal^24^,^26^ and in individuals receiving an HBIG-free entecavir regimen.^19^ One of the authors (R.P., unpublished observation, 2011) also observed this in a patient who was maintained on HBIG but had to undergo a temporary dose adjustment of entecavir because of calcineurin inhibitor toxicity. The long-term significance of this type of finding remains unclear. Although more is known the authors propose that a better term for the reappearance of HBsAg in the absence of detectable HBV DNA is *nonviremic HBsAg recurrence* rather than *recurrent infection*.

The frequency and nature of the adverse events and serious adverse events were consistent with those expected for this population. All serious adverse events were considered unrelated to the study treatment, and they were expected complications in posttransplant patients (either the result of preexisting CHB comorbidities or postoperative complications of antirejection therapy). Throughout the study, 15 events that met the definition of liver disease progression were reported. Fourteen of these events occurred within 30 days of transplantation and were considered to be due to postoperative complications. There have been reports of lactic acidosis in patients receiving entecavir with advanced liver disease and high Model for End-Stage Liver Disease scores.^27^–^29^ There were no reports of lactic acidosis for the patients in this study, although lactate levels were not systematically measured. Among the 24 patients who had evidence of HCC in the liver at the time of transplant, only 1 patient (4.2%) had established HCC recurrence. The observed rate of liver rejection in the present study (27.7%) is comparable to the rate reported elsewhere for combined NUC and HBIG therapy.^30^ Five patients died during the study. No death was considered related to the study treatment.

The results of this study are consistent with previous data demonstrating potent viral suppression and a favorable safety profile with entecavir in CHB patients with compensated or decompensated liver disease.^11^,^14^,^15^,^31^ Efficient viral suppression was observed throughout the present study, and no patient had virological recurrence (defined as a serum HBV DNA level ≥ 50 IU/mL). The results also confirm and extend findings from previous studies of entecavir use after HBV-related LT. Two small studies compared the efficacy of entecavir with the efficacy of lamivudine (both combined with long-term, low-dose HBIG). In both studies, among the patients treated with entecavir and HBIG, no case of HBV virological recurrence was observed after LT, whereas among patients treated with lamivudine and HBIG, 11% and 4% experienced HBV recurrence.^18^,^21^ In another study assessing an HBIG-free entecavir regimen,^19^ 79 of 80 patients (98.8%) had HBV DNA levels < 35 copies/mL, and 18 patients (22.5%) were HBsAg^+^ at the time of last follow-up (median duration = 26 months). Among the 18 HBsAg^+^ patients, all but 1 had undetectable HBV DNA at the time of last follow-up with no evidence of entecavir resistance. Thus, treatment with entecavir monotherapy after HBV-related LT is efficient in suppressing viral replication, although the rate of nonviremic HBsAg recurrence appears to be higher than the rate when it is combined with HBIG. Similar observations have also been observed with the maintenance of tenofovir/emtricitabine after HBIG withdrawal.^26^ Further follow-up studies are required to provide data on the long-term risk to the graft with HBIG-free antiviral regimens.

In the present study, the vast majority of patients were treated with entecavir combined with HBIG, which was mostly administered at a low dose. There was no difference in the treatment efficacy whether high-dose or lower dose HBIG was used with entecavir in this population of patients. This may have been partly because eligible patients were required to have low HBV DNA levels (<172 IU/mL) before entry. Patients with lower levels of serum HBV DNA before transplantation have a lower rate of recurrence, even with HBIG monotherapy.^32^–^34^ Furthermore, several studies have shown that patients with low-level viremia at the time of transplantation can be successfully protected from recurrent infection (HBsAg recurrence and detectable HBV DNA) despite HBIG withdrawal when they are treated with a combination of lamivudine and adefovir or tenofovir/emtricitabine.^24^,^26^

It should be noted that the current study had several limitations. The study involved a relatively short duration (72 weeks) of treatment, and it is possible that a longer duration of treatment would have shown a higher virological breakthrough rate in lamivudine-pretreated patients. The primary endpoint was assessed on the basis of an increase in the viral load, whereas HBV recurrence is commonly measured as the reappearance of HBsAg and detectable HBV DNA after LT. Inclusion criteria in this study required low HBV DNA levels at the baseline visit, and this patient population has been reported to have a lower risk for recurrent infection. Also, patients were treated with a multitude of HBIG regimens, which reflected different institutional policies. Despite these seeming limitations, the current study adds useful data because it provides proof that entecavir, an antiviral agent with a high genetic barrier, provides continuous viral suppression independent of the way in which HBIG is given, and it continues to protect the allograft from recurrent hepatitis even when HBsAg reappears. It is generally accepted that increased levels of HBV DNA after transplantation are a risk factor for graft failure and reduced survival.^28^ This fact, combined with the lack of recurrent hepatitis in the 2 patients with HBsAg reappearance observed in the present study, strongly suggests that undetectable HBV DNA rather than the reappearance of HBsAg is the best endpoint for which to aim after transplantation.

Further studies are needed to assess the efficacy and safety of entecavir with and without HBIG in patients undergoing LT. Recent studies have demonstrated that patients do not require HBIG therapy indefinitely if they are receiving potent antivirals with a high genetic barrier to resistance. Thus, studies looking at entecavir with low-dose intramuscular HBIG given for a definable period or without any HBIG in select patients would be particularly interesting and have important implications for reduction of cost.

## Abbreviations

ALT: alanine aminotransferase
CHB: chronic hepatitis B
HBeAb: hepatitis B e antibody
HBeAg: hepatitis B e antigen
HBIG: hepatitis B immune globulin
HBsAb: hepatitis B surface antibody
HBsAg: hepatitis B surface antigen
HBV: hepatitis B virus
HCC: hepatocellular carcinoma
LT: liver transplantation
NUC: nucleos(t)ide analogue
